# The 5Cs of Positive Youth Development, Purpose in Life, Hope, and Well-Being Among Emerging Adults in Malaysia

**DOI:** 10.3389/fpsyg.2021.641876

**Published:** 2021-07-15

**Authors:** Nor Ba’yah Abdul Kadir, Rusyda Helma Mohd

**Affiliations:** ^1^Psychology Program, Center for Research in Psychology and Human Well-being, Faculty of Social Sciences and Humanities, Universiti Kebangsaan Malaysia, Bangi, Malaysia; ^2^Human Development Program, Center for Research in Psychology and Human Well-being, Faculty of Social Sciences and Humanities, Universiti Kebangsaan Malaysia, Bangi, Malaysia

**Keywords:** positive youth development, 5Cs, well-being, emerging adults, Malaysia

## Abstract

A substantial body of evidence supports Lerner and colleagues’ 5Cs model of positive youth development (PYD) in the United States (U.S.). Nonetheless, it remains unclear whether the 5Cs can be used to identify positive development in the under-researched Asian contexts, such as Malaysia. Thus, this study examined the 5Cs of PYD (competence, confidence, character, connection, and caring) and their importance to purpose in life, hope, and well-being in a sample of emerging adult undergraduate university students in Malaysia. Data were collected from 400 participants from 15 Malaysian universities (132 males, 268 females; ages ranged from 18 to 26 years old, *M* = 22). A hierarchical multiple regression analysis indicated that two of the 5Cs of PYD (confidence and connection) as well as hope were important to explaining variation in well-being. The findings imply that there are strong links between PYD, especially confidence and connection, and well-being, while purpose in life and hope were indirectly related to the 2Cs (confidence and connection) of PYD and well-being. Therefore, mental health professionals are encouraged to review and redefine their treatment design to include confidence, connection, purpose in life and hope when working with Malaysian emerging adult university students.

## Introduction

Positive youth development (PYD) asserts that basic psychosocial conditions are significant determinants of youth well-being (Lerner, 2009). Positive youth development is based on the relational developmental systems theory, which suggests that young people possess resources that can be developed, nurtured, and cultivated (Lerner, 2009). A major PYD resource is the social context in which youths live such as the family, school, and community organizations. Lerner (2009) described PYD as a process that promotes the “5Cs”: competence, confidence, connection, character, and caring. Lerner (2009) also described thriving young people as individuals who actively nurture, cultivate, and develop positive qualities. In the 5Cs model, competence is the ability and skill to deal with the challenges, tasks, and stresses in life (Lerner, 2009). Besides that, confidence is a positive belief in one’s own worth and efficacy, while the term ‘connection’ describes positive relationships with others, including family members, peers, and communities (Lerner, 2009). Additionally, character defines standards of behavior that promote social functioning in societies (Lerner, 2009). Finally, caring implies a sense of sympathy and empathy for others (Lerner, 2009). The positive nature of the factors of PYD necessitates a strength-based approach rather than a deficit-based approach which is characterized by researchers concentrating on risk reduction to create more favorable growth conditions (Lerner et al., 2005). Positive youth development does not focus only on the development of individual strengths, but also devotes significant efforts at supporting positive relations between young people and their social-community resources. Beyond the 5Cs, other internal strengths of youth include purpose in life and hope. Hence, a variety of strengths and a positive identity, particularly purpose in life, may help emerging youths to not only adapt but also flourish as they enter the next phase of life (e.g., marriage, occupation).

### Well-Being as a Dimension of Mental Health

Based on Keyes (2002) model, one can expect possible positive associations between the 5Cs of PYD and well-being as a part of mental health. Specifically, Keyes (2002) created a fourfold classification system of mental health based on well-being, in which young people who score high on well-being and low on mental illness are flourishing; young people who score high on well-being and high on mental illness are struggling; young people who score low on well-being and high on mental illness are floundering; and young people who score low on well-being and low on mental illness are languishing. In this way, a concern for others and oneself are necessary to achieve the most lasting form of mental health. Thus, mental health is a complete state that consists of the absence of mental illness and the presence of a high-level of well-being (Keyes, 2002). This model puts forward the idea that mental health is multi-faceted and combines emotional well-being, psychological well-being, and social well-being, as well as the absence of recent mental illness.

Thus, young people who are mentally healthy are those who exhibit emotional vitality (e.g., happiness and life satisfaction), function well psychologically and socially, and are free of current and recent (i.e., 12 months) mental illness. The World Health Organization [World Health Organization [WHO], 2014, p. 12] defined mental health as “a state of well-being in which the individual realizes his or her abilities, can cope with the normal stresses of life, can work productively and fruitfully, and can make a contribution to his or her community.” This definition focuses on individual strengths (competence, confidence, connection, character, caring, hope) and a positive identity (purpose in life), and highlights the importance of “doing what is worth doing” (Ryan and Deci, 2001, p. 145) and the ability to accept challenges to achieve future goals (Tengland, 2001). This definition is aligned with that of Keyes (2002), who proposed that mental health is a multidimensional construct that includes well-being.

Indicators of mental health have been identified in several studies (Keyes, 2002; Korkeila et al., 2003; Farias et al., 2013; Orpana et al., 2016; Kotera and Ting, 2021). Orpana et al. (2016), for instance, identified 25 indicators of mental health for children, youth, and adults. Individual indicators include resilience, control, coping, violence, health status, physical activity, nurturing childhood, substance use, and spirituality. Additionally, family indicators comprise relationships, parenting style, health status, household composition, income, and substance use (Orpana et al., 2016). Furthermore, community indicators consisted of community involvement, social networks, social support, school, workplace, neighborhood, and social and built environment as well as community indicators such as inequality, political participation, discrimination, and stigma (Orpana et al., 2016). Less research on this topic has been conducted in Malaysia, however, a study in Malaysia examined associations among four indicators of mental health among university students, specifically, engagement, motivation, self-compassion, and well-being (Kotera and Ting, 2021). These constructs are not measured in this study but this study does show the need to examine well-being as a part of mental health in Malaysia.

Throughout the past decades, most researchers focused on exploring and determining mental health using measures of mental disorders (Vaillant, 2012). Until recently, studies linking the 5Cs of PYD and mental health have been relatively scarce compared to studies about mental disorders. In the PYD field, a great deal of research attention has been spent on studies that examine the psychometric properties of the 5Cs of PYD (Chen et al., 2018; Dvorsky et al., 2019), positive development in relation to positive and negative mental health (Holsen et al., 2017; Zhu and Shek, 2020), the development of instruments to be adapted into local contexts (Chai et al., 2020), and the effectiveness of interventions using a positive development approach (Ciocanel et al., 2017; Smith et al., 2018). Despite this diverse range of research, the exact associations between positive development, the 5Cs in particular, and mental health are still being examined, with little of this research having been conducted in Malaysia.

### Purpose in Life in Relation to Other Study Constructs

Purpose in life is associated with mental health (Van Dyke and Elias, 2007). Various conceptualizations of purpose in life are well-established in the existing literature. Purpose in life refers to “a central, self-organizing life aim that organizes and stimulates goals, manages behaviors, and provides a sense of meaning” (Kashdan and McKnight, 2009, p. 304). According to Kashdan and McKnight (2009), it is central in that it has to do with a person’s identity, and it is self-organizing in that it provides a framework for systematic behavior in daily life. Purpose in life may motivate people to plan and make efforts to achieve a specific goal, and it helps them to make decisions and perform specific behaviors to proceed in life (Hill et al., 2016; Zhang et al., 2018). However, the existing literature regarding young people defines purpose in life as a “stable and generalized intention to accomplish something that is at once meaningful to the self and of consequence to the world beyond the self” (Damon and Gregory, 2003, p. 121).

Purpose in life can also act as a protective factor in relation to mental health (Galek et al., 2015; Aghababaei et al., 2016; Glaw et al., 2017), well-being (Stoyles et al., 2015), and happiness (Aghababaei and Błachnio, 2014). Those who exhibit a strong sense of purpose in life also express greater self-esteem (Błażek and Besta, 2012) and happiness (Crego et al., 2021), and less depression (Hartanto et al., 2020). In a study carried out among Chinese emerging adults, Zhang et al. (2018) examined the effects of purpose in life with various variables in relation to mental health. The results revealed that purpose in life has a significant, negative association with stress, anxiety and depression, and a significant, positive association with gratitude, school belonging, and grit. Bronk et al. (2010) found the prevalence rates of purpose in life among high-ability early adolescents and high-ability late adolescents were roughly the same. Furthermore, Aghababaei et al. (2016), in their study among Iranian university students, found that purpose in life was a significant predictor of subjective well-being. A study conducted among emerging adults by Bronk et al. (2009) confirmed that there was a positive relation between purpose in life and life satisfaction. In a study conducted on adolescents in Central Israel, two types of purpose in life were examined. The study found that both types of purpose in life had a higher life satisfaction (Blau et al., 2019). Thus, the current research literature shows that there should be a modest relation between purpose in life and well-being. However, we were not able to locate any published studies concerning the 5Cs of PYD and purpose in life.

### Hope in Relation to Other Study Constructs

Similar to purpose in life, hope also plays a significant role in PYD. It has been identified as a character strength (Preacher and Hayes, 2008; Lopez and Snyder, 2005). Like purpose in life, the hope construct focuses on significant future aims. Hope consists of an element that involves the motivation of an individual to pursue his or her goals. Thus, hope refers to the perceived ability and capacity of an individual to achieve future goals through mental energy and to generate routes toward those goals (Belen et al., 2020). Therefore, hope sees potential pathways to the achievement of desired goals, and it inspires a person to use those pathways (Rand and Cheavens, 2009; Du et al., 2016; Tirrell et al., 2019). Hope consists of motivation (agency thinking) and confidence (pathway thinking), which may spark individuals to pursue goals and produce strategies to achieve the desired goals (Yalçın and Malkoç, 2015; Guse and Shaw, 2018). In other words, hope is the result of believing that realistic plans can be created, and having sufficient drive to achieve important goals. Thus, individuals with mental health might develop hope, agentic, and pathways thinking. The latter definition is believed to capture the essence of that which is involved in hopeful, goal-directed thoughts, and at the same time, it is consonant with the everyday understanding of the term. Therefore, this study relied on this definition of hope.

Hope may also play a vital role in setting one’s purpose in life, thereby, potentially supporting well-being (Ciarrochi et al., 2007; Burrow et al., 2010; Parker et al., 2015; Årdal et al., 2018). Hope is also related to various factors such as life satisfaction (Munoz et al., 2017), resilience (Li et al., 2016; Lenz, 2021), academic achievement (Marques et al., 2011, 2017; Bryce et al., 2020), and subjective well-being (Zhang and Chen, 2018). Further, in the existing literature, significant associations have been found between hope, emotional well-being (Griggs and Crawford, 2017), and psychological well-being (Dilmaç and Kocaman, 2019). To illustrate this point, in a cross-sectional study of 495 college students, after controlling for gender, race, age, and social desirability, a strong linear positive relation existed between hope and emotional well-being (Griggs and Crawford, 2017). Also, Gallagher et al. (2021) found that hope significantly acted as a proactive factor during crisis and improved well-being. We located only one published study that examined the relations between hope and purpose in life in a sample of adolescents and emerging adults (Bronk et al., 2009). Therefore, more work is needed to understand these relations.

It is necessary to understand the associations between the 5Cs of PYD and well-being as a facet of mental health, as well as conceptually connected but distinct positive constructs such as purpose in life and hope. However, no study has comprehensively explored the relations between the 5Cs of PYD, purpose in life, hope, and mental health with Malaysian youth or emerging adults. Furthermore, there has been no in-depth investigation into how strongly each factor is related to mental health. Thus, this study, is a first of its kind investigation into the possible relations between the 5Cs of PYD, purpose in life, hope, and well-being as a facet of mental health among Malaysian emerging adults. The following hypotheses and one research question were examined:

H_1_: The 5Cs of PYD are associated with well-being.
H_2_: Purpose in life is associated with well-being.
H_3_: Hope is associated with well-being.

RQ_1__:_ How much of the variance in well-being is explained by the 5Cs, purpose, and hope.

H_4_: Purpose has an indirect association with the 5Cs of PYD and well-being.
H_5_: Hope has an indirect association with the 5Cs of PYD and well-being.

## Materials and Methods

### Settings and Participants

Participants were 400 undergraduate students from 15 universities in Malaysia. Each university provided a minimum of 20 participants that completed an online survey. Out of the 15 universities, 13 were located in Peninsular Malaysia, one in the Northwest (Sarawak) and one in Northern Borneo Island (Sabah). The participants ranged in age from 18 to 26 years old (*M* = 21.50, *SD* = 1.21, 66.8%, *n* = 263 females). The participants identified themselves as Muslims (79.8%, *n* = 315), followed by Hindus (7.4%, *n* = 29), Buddhists (6.3%, *n* = 25), Catholics (3%, *n* = 12), Pentecostals (1.5%, *n* = 6), Protestants (1%, *n* = 4), followers of Kong Hu Chu (0.5%, *n* = 2), and Mysticism (0.3%, *n* = 1). Most of the participants (88.6%, *n* = 349) agreed completely that religion was important in their lives. A total of 75.9% (*n* = 299) of the participants were living with their parents, while the rest were living with their guardians (5.8%, *n* = 23), living with their mother only (8.6%, *n* = 34), living with their father only (1.5%, *n* = 6), living alone (4.8%, *n* = 19), or were taking turns living with their father or mother (3.3%, *n* = 13). Around 13.7% (*n* = 54) rated their academic achievement as excellent and 29.9% (*n* = 118) rated it as very good.

This study was part of a larger cross national research study located at the University of Bergen, Norway. The larger study concerns PYD and involves over 30 countries. Thus, this study, with its individual study protocols specifying the aggregation of data across sites for analysis and dissemination, was approved by the Institutional Review Board (IRB) of the University of Bergen. It was implemented in accordance with the guidelines stated in the Declaration of Helsinki. An online version of this survey was administered to the participating undergraduate students. The universities selected were those that were most easily accessible to the research team, and therefore, the student participants comprised a convenience sample.

Before completing the online survey, the participants were given a brief introduction to the purpose and aims of this study. Each participant took approximately 40–45 min to complete the online questionnaire. This online survey data collection involved a sample of volunteers who were interested in taking the survey; therefore, it was impossible to calculate the number of participants who decided not to respond. This was a one-time data collection and thus the study design was cross-sectional.

### Measures

Two bilingual speakers translated the questionnaire from English to Malay and, at the same time, adhered to the cross-cultural translation standard requirements proposed by Beaton et al. (2000). One translator was aware of the PYD concepts, while another translator was neither informed nor aware of the PYD concepts and had no background in developmental psychology. The translators and researchers then discussed the discrepancies in the items, and the wordings were changed for any disputed items. Then, a pilot test was conducted among 30 students to determine if the items were equivalent. The final Malay version of the questionnaire was then utilized in this study.

#### Demographic Questions

Participants were asked several demographic questions such as their gender, ethnicity, age, religion and its importance in their lives, current living arrangements, and self-reported academic achievement. Example of question on current living arrangements: “Who do you live with?” (e.g., mother and father, mainly with mother, mainly with father, as much with mother as with father, with adults who are not my parents, live alone). Example of question on academic achievement: “How would you rate your academic performance?” (5-point Likert scale from poor to excellent).

#### The 5Cs

The 5Cs of PYD scale-short form (Geldhof et al., 2014) was used to measure PYD. The PYD-5C is a self-report measure consists of 34 items that serve as indicators for each of the 5Cs (competence, confidence, character, connection, caring). The scores for each PYD construct were calculated as mean scores with high scores indicating high levels of each C. In this study, all the Cronbach’s alpha values for these subscales were satisfactory (competence, α = 0.86; confidence, α = 0.91; character, α = 0.80; caring, α = 0.91; connection, α = 0.88).

#### Well-Being as an Index of Mental Health

The Adolescent Mental Health Continuum-Short Form (Keyes, 2002, 2009) was used to measure how often a positive mental health event occurred within the past month. The original 14-item Mental Health Continuum-Short Form (MHC-SF; Keyes, 2005) consists of three items measuring emotional well-being, five items measuring social well-being, and six items measuring psychological well-being. Emotional well-being refers to positive emotions, while social well-being refers to social contribution, social integration, social actualization, social acceptance, and social coherence. Psychological well-being refers to self-acceptance, environmental mastery, positive relations with others, personal growth, autonomy, and purpose in life. The participants were asked to respond to items on a 6-point Likert-type scale based on their experience over one month. Each response referred to: never, once or twice, about once a week, 2 or 3 times a week, almost every day, and every day. In this study, the value of the Cronbach’s alpha for MHC-SF was satisfactory (α = 0.96). For this study, the MHC-SF total score was calculated as an index of overall mental health. Thus, a higher score would indicate a higher level of positive mental health.

#### Purpose in Life

This study used a brief measure of purpose in life developed by Hill et al. (2016), which consists of four items (5-point Likert scale). Examples of these items are: “There is a direction in my life,” “My plans for the future match my true interests and values,” “I know which direction I am going to follow in my life,” and “My life is guided by a set of clear commitments.” Summative scores ranged from 4 to 20, with a higher total score denoting greater purpose in life. The Cronbach’s alpha for this measure in the present study was.91.

#### Hope

The Herth Hope Index [(HHI); Herth, 1992] is an adapted version of the Herth Hope Scale, and it conceptually addresses the four attributes of hope described in the Hope Process Framework (Farran et al., 1995). Originally, it is a 12-item (5 point) Likert scale that delineates three subscales of hope: (1) temporality and future, (2) positive readiness and expectancy, and (3) interconnectedness. In the present study, the item “I am able to give and receive caring/love” was separated into two items: (1) I am able to give caring/love, and (2) I am able to receive caring/love. Summative scores ranged from 13 to 65, with a higher total score denoting greater hope. The Cronbach’s alpha for the HHI in the present study was.88.

### Data Analysis

The distribution and descriptive analysis statistics were obtained through the Statistical Package for the Social Sciences (SPSS) version 26. The data were screened for outliers and assumptions for parametric tests. Pearson’s product-moment correlation coefficients were used to explore the associations between variables. Then, a multiple regression was performed to determine how much variance could be explained by each variable. The PROCESS macro for the SPSS, model 4 with bootstrapping – a resampling procedure to avoid forcing the assumption of normality for the sampling distribution of the indirect effect – was utilized testing hypotheses four and five regarding indirect associations (Preacher and Hayes, 2008). For this analysis, a sampling distribution was generated with 95% confidence intervals to test for indirect associations, which were considered significant if zero did not fall between the upper and lower confidence intervals (Preacher and Hayes, 2008). In this study, the bootstrapped confidence intervals for the indirect associations were based on 10,000 resamples.

## Results

### Associations Between the 5Cs, Purpose in Life, Hope, and Well-Being

No outliers were identified using the outlier labeling rule (Hoaglin and Iglewicz, 1987). The bivariate Pearson’s correlations were calculated to explore the relations between the 5Cs of PYD, purpose in life, hope, and well-being (See Table 1). All the study variables were significantly correlated with each other in the expected directions. Based on the rule of thumb by Hair et al. (2014), correlations between all the variables showed relations ranging from weak to moderate. Regarding hypothesis 1, a significant positive association was obtained between the 5Cs of PYD and well-being (competence: *r* = 0.58, *p* < 0.01; confidence: *r* = 0.71, *p* < 0.01; character: *r* = 0.56, *p* < 0.01; caring: *r* = 0.36, *p* < 0.01; connection: *r* = 0.67, *p* < 0.01). These positive coefficients indicated that when the value of one variable increased, the values of the other variables also tended to increase.

**TABLE 1:** Descriptive statistics and correlations between the study constructs (*N* = 393).

Variables	M	SD	1	2	3	4	5	6	7	8
1. Well-Being	4.48	1.02	–							
2. Competence	3.34	0.81	0.582**	–						
3. Confidence	3.64	0.83	0.713**	0.711**	–					
4. Character	3.85	0.62	0.556**	0.522**	0.579**	–				
5. Caring	4.10	0.74	0.362**	0.203**	0.289**	0.654**	–			
6. Connection	3.74	0.70	0.609**	0.532**	0.536**	0.545**	0.455**	–		
7. Hope	3.82	0.61	0.603**	0.370**	0.525**	0.452**	0.368**	0.418**	–	
8. Purpose in Life	3.93	0.79	0.589**	0.487**	0.617**	0.604**	0.413**	0.607**	0.494**	–

** p < 0.01 (2-tailed)

Similarly, in regards to hypothesis 2, a significant positive relation was obtained between purpose in life and well-being (*r* = 0.59, *p* < 0.01). This relation showed that as the value of purpose in life increased, well-being also tended to increase. In regards to hypothesis 3, a significant positive relation was obtained between hope and well-being (*r* = 0.60, *p* < 0.01). This relation showed that as hope increased, well-being tended to increase. The inter-correlation of the variables that were studied revealed that the variables did not overlap. Oxford and Burry-Stock’s (1995) recommendations were used to determine the mean score levels (high: 3.5–5.0; moderate: 2.5–3.4; low: 1.0–2.4). Thus, the mean scores for the 5Cs of PYD, hope, and purpose in life were considered to be high. However, the mean score for competence was moderate.

Several requirements and statistical assumptions were examined before a multiple regression analysis was performed. These requirements and assumptions comprised sample size and normality, homoscedasticity, linearity, multicollinearity, and outliers. Consequently, it was found that the sample size fulfilled the minimum requirement. The present study included eight main variables: confidence, competence, connection, character, caring, purpose in life, hope, and well-being. In addition, the sample size of 400 was sufficient according to the recommendations provided by Knofczynski and Mundfrom (2008). The assumption of a normal distribution was also fulfilled based on a graphical examination, which showed that the data were normally distributed. The values of skewness and kurtosis also satisfied the minimum requirement (See Table 2). By evaluating the scatterplot, the presumption of homoscedasticity revealed that the variances were equivalent. An examination of the scatterplot graph showed that the linearity assumption was met as the predictor and criterion data were distributed along a linear line. The casewise diagnostics was also examined to determine which cases included residuals and which ones were three or more standard deviations away from the mean. Hence, only 393 cases were used in the analysis connected to Research Question 1. Singularity was not violated as the variables for the predictors included independent and unique variables that did not overlap. Multicollinearity tests were also carried out to avoid possible problems of overlapping between variables. Because all the indices of tolerance were greater than 1-*R*j2, no variables in the study were removed.

**TABLE 2:** Skewness and kurtosis values of the study constructs.

Variables	Skewness	Kurtosis
Well-Being	–0.570	–0.377
Hope	–0.899	1.394
Purpose in life	–0.576	0.195
Competence	–0.107	–0.377
Confidence	–0.694	0.361
Character	–0.701	1.020
Caring	–0.921	0.710
Connection	–0.535	0.404

Following the correlation analysis, a hierarchical regression analysis (stepwise) was performed to determine the importance of confidence, competence, connection, character, caring, purpose in life, and hope on well-being (Table 3) as a test of Research Question 1. Gender (male, female), ethnicity (Malay, non-Malay), age, academic performance, and either the participants lived with their parents or mother only or father only or alone were treated as covariates. Dummy variables were created for gender and ethnicity. Because of the explorative nature of this research question, variables with non-significant effects were removed one by one stepwise. Gender, ethnicity, age, academic performance, and participants’ living arrangements were entered in Step 1. In Step 2, confidence, competence, connection, character, caring, purpose in life, and hope were included.

**TABLE 3:** Hierarchical regression analysis (Stepwise): Well-Being in Relation to 5Cs of PYD, purpose in life, and hope (*N* = 393).

Factors	Beta	t	Sig.	Collinearity statistics	
				Tolerance	VIF
**Model 1**					
Constant		33.955	0.000***		
Academic performance	–0.245	–5.007	0.000***	1.000	1.000
**ΔR^2^ = 0.058; F_(__1_, _391__)_ = 35.073, p < 0.001**					
**Model 2**					
Constant		31.807	0.000***		
Academic performance	0.160	–4.796	0.000***	0.991	1.009
Male	0.112	2.285	0.023*	0.991	1.009
**ΔR^2^ = 0.068; F_(__2_, _390__)_ = 15.283, p < 0.001**					
**Model 3**					
Constant		6.492	0.000***		
Academic performance	–0.049	–1.322	0.187	0.920	1.088
Male	0.059	1.663	0.097	0.985	1.015
Confidence	0.693	18.801	0.000***	0.918	1.089
**ΔR^2^ = 0.510; F_(__3_, _389__)_ = 137.229, p < 0.001**					
**Model 4**					
Constant		0.674	0.501		
Academic performance	–0.045	–1.307	0.192	0.919	1.088
Male	0.055	1.669	0.096	0.985	1.016
Confidence	0.536	13.784	0.000***	0.684	1.463
Connection	0.318	8.223	0.000***	0.724	1.382
**ΔR^2^ = 0.628; F_(__4_, _388__)_ = 136.993, p < 0.001**					
**Model 5**					
Constant		–2.031	0.043*		
Academic performance	–0.020	–0.610	0.542	0.909	1.101
Male	0.059	1.894	0.059	0.984	1.016
Confidence	0.421	10.438	0.000***	0.583	1.714
Connection	0.264	7.164	0.000***	0.698	1.434
Hope	0.265	7.097	0.000***	0.679	1.473
**Δ*R*^2^ = 0.630; *F*_(__5_, _387__)_ = 133.612, *p* < 0.001**					

*** p < 0.001; *p < 0.05.

The hierarchical regression produced five models (See Table 3). The final model (model 5) indicated that the variables of confidence, connection, and hope significantly added to the prediction. An examination of the standardized regression coefficients revealed that confidence (β = 0.421, *p* < 0.001), hope (β = 0.264, *p* < 0.001), and connection (β = 0.265, *p* < 0.001) were significantly associated with well-being after accounting for gender, ethnicity, age, academic performance, and participants living arrangements. The interpretation, therefore, was that changes in confidence, connection, and hope were positively related to changes in well-being. The final regression model explained 63% of the variance in well-being. These results indicated that confidence, connection, and hope were important to explaining the variance in well-being. Because the associations of competence, character, and caring in relation to well-being not significant, these factors were excluded from the analysis related to Hypotheses 4 and 5.

### Test of Indirect Associations

**FIGURE 1:**
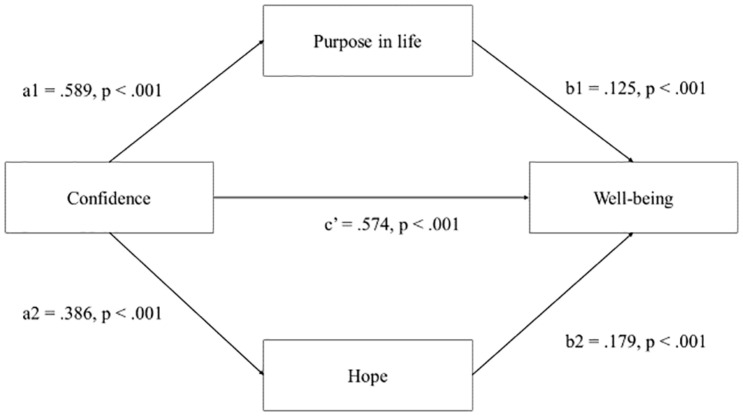
Test of indirect associations for purpose in life and hope in relation to confidence and well-being.

**FIGURE 2:**
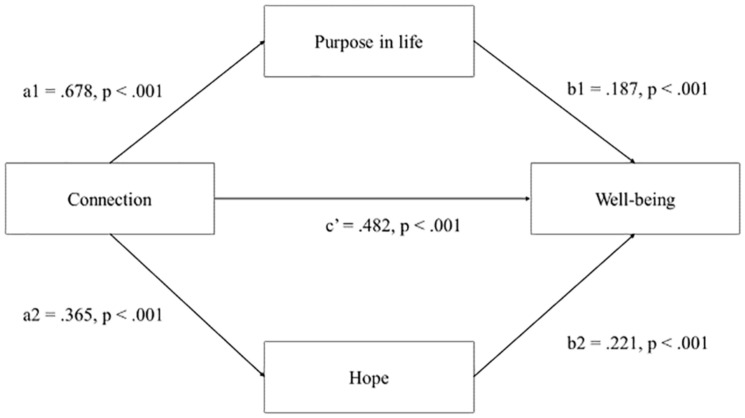
Test of indirect associations in regards to purpose in life and hope as indirectly associated with connection and well-being.

To test hypotheses 4 and 5, two sets of analyses were conducted using model number 4 in the PROCESS macro (Hayes, 2017) to test for the possibility of indirect effects (i.e., associations) in regards to purpose in life and hope as indirectly associated with confidence and connection in PYD and well-being. The direct effect of confidence on well-being was significant [*b* = 0.574, *t* = 10.757, *p* < 0.001, CIs (0.4694,0.6793)]. The results also showed that confidence in PYD was indirectly associated with well-being through its association with purpose in life and hope. It can be seen in Figure 1 that the indirect effect of confidence on well-being through purpose in life [*b* = 0.125, CI (0.0486,0.2083)] and hope [*b* = 0.179, CI (0.1019,0.2698)] was significant. Hence, purpose in life and hope appeared to have significant indirect associations with confidence and well-being (Figure 2).

The possibility of indirect associations in regards to purpose in life and hope in relation to connection in PYD and well-being was also tested in the PROCESS macro (Hayes, 2017). The direct association or effect of connection on well-being was significant [*b* = 0.482, *t* = 7.5228, *p* < 0.001, CIs (0.3564,0.6086)]. The indirect effect of connection on well-being via purpose in life [*b* = 0.187, CI (0.0856,0.2946)] and hope [*b* = 0.221, CI (0.1360,0.3185)] was also significant. Hence, purpose in life and hope had significant indirect associations with connection and well-being.

## Discussion and Conclusion

This study was aimed at investigating the associations among the 5Cs of PYD, purpose in life, hope, and well-being as a facet of mental health in a university-based sample of Malaysian emerging adults. This study was also examined the roles of purpose in life and hope as potentially having indirect associations with some of the 5Cs of PYD and well-being. Consistent with the first hypothesis, the results indicated that all the 5Cs of PYD were significantly associated with well-being. Some outcomes were consistent with previous studies in that 2Cs out of the 5Cs (confidence, connection) might have both direct and indirect effects on well-being through purpose in life and hope.

As expected by Conway et al. (2015), it was discovered in this study that the 5Cs of PYD, namely competence, confidence, connection, character, and caring, had a significant and positive correlation with well-being as a facet of mental health. These findings were consistent with those of previous studies (e.g., Årdal et al., 2018). These results seem to suggest that as the levels of competence, confidence, connection, character, and caring increase, so does well-being and vice versa. Going back to PYD theory, this can be explained in terms of the developmental relational systems theory that the 5Cs of PYD are important to other aspects of healthy or positive development. Furthermore, it was also found that purpose in life and hope had a significant and positive correlation with the 5Cs, and this was consistent with a previous study by Bronk et al. (2009). In Malaysia, few studies have been conducted to examine such associations. However, previous findings have shown that the relation between PYD and well-being is mostly positive (e.g., Park, 2004). The results of this study revealed that 2Cs (confidence, connection) were important factors, and had a significant and positive direct association with well-being. The term ‘confidence’ can be explained as a belief in someone’s ability to accomplish goals, form connections with others in various situations, and overcome many obstacles. As a result, it should have a meaningful impact on life. Thus, the ability to initiate connections with others such as peers, family members, neighbors, and communities reflects a higher level of confidence among emerging adults. Furthermore, establishing connections with others may help emerging adults to improve their lives and the lives of those around them.

As hypothesized earlier, it was discovered that purpose in life was indirectly associated with confidence and well-being. In particular, it appeared that the link between confidence and well-being was indirectly affected by purpose in life. Purpose in life involves motivation (Bronk et al., 2010) and may have implications for emerging adults in developing confidence. For instance, emerging adults may build their confidence when they actively search for and develop a purpose in life after they have learnt to do so from the behavior of others (Kashdan and McKnight, 2009). Formulating short-term and long-term goals in life, for instance, may encourage emerging adults to be prospective and future oriented, and goals could include securing decent jobs, professional success and having families.

As hypothesized, it was also discovered that purpose in life was indirectly associated with connection and well-being. It appeared that the link between connection and well-being was indirectly connected with purpose in life. This finding suggests that connections may be developed when emerging adults experience supportive relationships with others are important. Hence, the results indicated that the link between connection and well-being via purpose in life may be an important factor for emerging adults to flourish and thrive.

Likewise, as hypothesized, it was found that hope was important to the relation between confidence and well-being. Specifically, it appeared that the link between confidence and well-being was indirectly connected to hope. This relation suggests that to improve well-being as a key part of well-being, involves supporting confidence and hope, as a way to motivate emerging adults to achieve their goals by determining which pathway they should take to accomplish them. Furthermore, hope plays a vital role during a crisis because it allows individuals to take further steps more confidently to strategize their plans and thus, accomplish their goals.

Also, as hypothesized, it was found that hope was an important to the relation between connection and well-being. This finding suggests that the relation between connection and well-being is also indirectly connected to hope. In the context of positive relationships with others, hope may help individuals to improve their relationships during difficult situations.

Despite the sufficient sample and the generally acceptable reliability of the scales used, several limitations of the present study should be noted. First, the sample was selected from undergraduate students and is not representative of the entire undergraduate university student population of Malaysia, and is not representative of all emerging adults in Malaysia such as those individuals who are not attending university. Although the level of cooperation was good, the study was lacking in information about those undergraduate students who did not respond. It is not known whether the sample was biased. Secondly, the cross-sectional design of the study, which stipulated that all measures were taken only once, means it was impossible to establish causal relationships, and the indirect associations found in this study are not indicative of statistical mediation which should be examined through future longitudinal research studies conducted with Malaysian university students.

This study demonstrated in this Malaysia sample the importance of 2Cs of PYD and the connection to purpose in life and hope as important to well-being. The main idea in helping emerging adults thrive and flourish is to systematically align their potential and strengths with contextual resources over time for healthy development, thus enabling them to develop cognitive, affective, and behavioral components concerning confidence and connection. Contextual resources were not systematically examined in this study, however, this is an important future research focus. This idea is crucial in observing some emerging adults who seem to be performing better in one particular area but may not be doing so in other aspects of their lives. The same goes for those who seem to be failing in one field but may not be performing poorly in other areas. Based on the findings in this study, it is believed that the 2Cs of PYD (connection, confidence), purpose in life, and hope will help emerging youths to thrive and flourish and to experience greater well-being as a fundamental part of mental health (Keyes, 2005).

Despite the limitations noted, this study makes a unique contribution to the existing knowledge about PYD, by conducting this study with emerging adults attending university in Malaysia. The findings, along with the findings of other future studies, start to provide a basis for developing intervention programs and as a reference for related research and a National Youth Policy in Malaysia. For instance, counselors in university settings are encouraged to consider specific activity programs to at least develop the 2Cs of PYD, and to pay close attention to purpose in life and hope, which can be achieved through a systematic intervention planning and strategy to increase the strengths of emerging adults, and, thereby, improve the mental health of university students.

To conclude, this study affirms that the 5Cs of PYD, particularly connection and confidence, are positively correlated with well-being and theoretically we viewed well-being as a key facet of mental health consistent with Keyes (2005). The results also highlight the importance of purpose in life and hope in connection with well-being. The results of this study can be used as a reference by other researchers who are interested in studying the 5Cs of PYD, purpose in life, and hope among Malaysian emerging adults and more research should be conducted on this topic in general population samples of emerging adults, adolescents, and youth and emerging adults in contact with psychiatric services. It will improve the understanding of other protective factors in increasing the well-being of emerging adults by exploring their strengths, purpose in life, and hope.

## Data Availability Statement

The raw data supporting the conclusions of this article will be made available by the authors, without undue reservation.

## Ethics Statement

The studies involving human participants were reviewed and approved by the Institutional Review Board (IRB) at the University of Bergen. The patients/participants provided their written informed consent to participate in this study.

## Author Contributions

Both authors listed have made a substantial, direct and intellectual contribution to the work, and approved it for publication.

## Conflict of Interest

The authors declare that the research was conducted in the absence of any commercial or financial relationships that could be construed as a potential conflict of interest. The action editor for this manuscript, LF-W is a contributing researcher to the wider cross-national project that this study is part of. There is no past, present, or planned future research collaborations between the study authors and the action editor of this manuscript.

## References

[B1] Aghababaei N. Błachnio A. (2014). Purpose in life mediates the relationship between religiosity and happiness: evidence from Poland. *Ment. Health Relig. Cult.* 17 827–831. 10.1080/13674676.2014.928850

[B2] Aghababaei N. Sohrabi F. Eskandari H. Borjali A. Farrokhi N. Chen Z. J. (2016). Predicting subjective well-being by religious and scientific attitudes with hope, purpose in life, and death anxiety as mediators. *Pers. Individ. Differ.* 90 93–98. 10.1016/j.paid.2015.10.046

[B3] Årdal E. Holsen I. Diseth Å Larsen T. (2018). The five Cs of positive youth development in a school context; gender and mediator effects. *School Psychol. Int.* 39 3–21.

[B4] Beaton D. E. Bombardier C. Guillemin F. Ferraz M. B. (2000). Guidelines for the process of cross-cultural adaptation of self-report measures. *Spine* 25 3186–3191. 10.1097/00007632-200012150-00014 11124735

[B5] Belen H. Yildirim M. Belen F. S. (2020). Influence of fear of happiness on flourishing: mediator roles of hope agency and hope pathways. *Aust. J. Psychol.* 72 165–173. 10.1111/ajpy.12279

[B6] Blau I. Goldberg S. Benolol N. (2019). Purpose and life satisfaction during adolescence: the role of meaning in life, social support, and problematic digital use. *J. Youth Stud.* 22 907–925. 10.1080/13676261.2018.1551614

[B7] Błażek M. Besta T. (2012). Self-concept clarity and religious orientations: prediction of purpose in life and self-esteem. *J. Relig. Health* 51 947–960. 10.1007/s10943-010-9407-y 20953709 PMC3444705

[B8] Bronk K. C. Hill P. L. Lapsley D. K. Talib T. L. Finch H. (2009). Purpose, hope, and life satisfaction in three age groups. *J. Posit. Psychol.* 4 500–510. 10.1080/17439760903271439

[B9] Bronk K. C. Holmes Finch W. Talib T. L. (2010). Purpose in life among high ability adolescents. *High Ability Stud.* 21 133–145. 10.1080/13598139.2010.525339

[B10] Bryce C. I. Alexander B. L. Fraser A. M. Fabes R. A. (2020). Dimensions of hope in adolescence: relations to academic functioning and well-being. *Psychol. Schools* 57 171–190. 10.1002/pits.22311

[B11] Burrow A. L. O’dell A. C. Hill P. L. (2010). Profiles of a developmental asset: youth purpose as a context for hope and well-being. *J. Youth Adolesc.* 39 1265–1273. 10.1007/s10964-009-9481-1 19937095

[B12] Chai X. Wang J. Li X. Liu W. Zhao G. Lin D. (2020). Development and validation of the Chinese positive youth development scale. *Appl. Dev. Sci.* 1–14. 10.1080/10888691.2020.1712206

[B13] Chen B. B. Wiium N. Dimitrova R. (2018). Factor structure of positive youth development: contributions of exploratory structural equation modeling. *Pers. Individ. Differ.* 124 12–15. 10.1016/j.paid.2017.11.039

[B14] Ciarrochi J. Heaven P. C. Davies F. (2007). The impact of hope, self-esteem, and attributional style on adolescents’ school grades and emotional well-being: a longitudinal study. *J. Res. Pers.* 41 1161–1178. 10.1016/j.jrp.2007.02.001

[B15] Ciocanel O. Power K. Eriksen A. Gillings K. (2017). Effectiveness of positive youth development interventions: a meta-analysis of randomized controlled trials. *J. Youth Adolesc.* 46 483–504. 10.1007/s10964-016-0555-6 27518860

[B16] Conway R. J. Heary C. Hogan M. J. (2015). An evaluation of the measurement properties of the five Cs model of positive youth development. *Front. Psychol.* 6:1941. 10.3389/fpsyg.2015.01941 26733923 PMC4686649

[B17] Crego A. Yela J. R. Gómez-Martínez M. Á Riesco-Matías P. Petisco-Rodríguez C. (2021). Relationships between mindfulness, purpose in life, happiness, anxiety, and depression: testing a mediation model in a sample of women. *Int. J. Environ. Res. Public Health* 18:925. 10.3390/ijerph18030925 33494471 PMC7908241

[B18] Damon W. Gregory A. (2003). “Bringing in a new era in the field of youth development,” in *Developmental Assets and Asset-Building Communities*, eds Lerner R. M. Benson P. L. (Boston, MA: Springer), 47–64. 10.1007/978-1-4615-0091-9_3

[B19] Dilmaç B. Kocaman E. N. (2019). Investigation of the relationship between hope and the psychological well-being in a group of adults in terms of different variables. *Soc. Sci. Educ. Res. Rev.* 6 6–29.

[B20] Du H. King R. B. Chu S. K. (2016). Hope, social support, and depression among Hong Kong youth: personal and relational self-esteem as mediators. *Psychol. Health Med.* 21 926–931. 10.1080/13548506.2015.1127397 26714559

[B21] Dvorsky M. R. Kofler M. J. Burns G. L. Luebbe A. M. Garner A. A. Jarrett M. A. (2019). Factor structure and criterion validity of the five Cs model of positive youth development in a multi-university sample of college students. *J. Youth Adolesc.* 48 537–553. 10.1007/s10964-018-0938-y 30298222 PMC6391208

[B22] Farias M. Underwood R. Claridge G. (2013). Unusual but sound minds: mental health indicators in spiritual individuals. *Br. J. Psychol.* 104 364–381. 23848387 10.1111/j.2044-8295.2012.02128.x

[B23] Farran C. J. Herth K. A. Popovich J. M. (1995). *Hope and Hopelessness: Critical Clinical Constructs.* New York, NY: Sage Publications.

[B24] Galek K. Flannelly K. J. Ellison C. G. Silton N. R. Jankowski K. R. (2015). Religion, meaning and purpose, and mental health. *Psychol. Relig. Spiritual.* 7 1–12.

[B25] Gallagher M. W. Smith L. J. Richardson A. L. D’Souza J. M. Long L. J. (2021). Examining the longitudinal effects and potential mechanisms of hope on COVID-19 stress, anxiety, and well-being. *Cogn. Behav. Ther.* 50 234–245. 10.1080/16506073.2021.1877341 33544032

[B26] Geldhof G. J. Boyd M. J. Mueller M. K. Napolitano C. M. Schmid K. L. Lerner J. V. (2014). Creation of short and very short measures of the five Cs of positive youth development. *J. Res. Adolesc.* 24 163–176. 10.1111/jora.12039

[B27] Glaw X. Kable A. Hazelton M. Inder K. (2017). Meaning in life and meaning of life in mental health care: an integrative literature review. *Issues Ment. Health Nurs.* 38 243–252. 27929687 10.1080/01612840.2016.1253804

[B28] Griggs S. Crawford S. L. (2017). Hope, core self-evaluations, emotional well-being, health-risk behaviors, and academic performance in university freshmen. *J. Psychosoc. Nurs. Ment. Health Serv.* 55 33–42. 10.3928/02793695-20170818-11 28850649

[B29] Guse T. Shaw M. (2018). “Hope, meaning in life and well-being among a group of young adults,” in *Hope for a Good Life*, eds Krafft A. Perrig-Chiello P. Walker A. (Cham: Springer), 63–77. 10.1007/978-3-319-78470-0_3

[B30] Hair J. F. Black W. C. Babin B. J. Anderson R. E. (2014). *Multivariate Data Analysis. Pearson New International Edition.* London: Pearson.

[B31] Hartanto A. Yong J. C. Lee S. T. Ng W. Q. Tong E. M. (2020). Putting adversity in perspective: purpose in life moderates the link between childhood emotional abuse and neglect and adulthood depressive symptoms. *J. Ment. Health* 29 473–482. 10.1080/09638237.2020.1714005 31983245

[B32] Hayes A. F. (2017). *Introduction to Mediation, Moderation, and Conditional Process Analysis: A Regression-Based Approach.* New York, NY: Guilford publications.

[B33] Herth K. (1992). Abbreviated instrument to measure hope: development and psychometric evaluation. *J. Adv. Nurs.* 17 1251–1259. 10.1111/j.1365-2648.1992.tb01843.x 1430629

[B34] Hill P. L. Edmonds G. W. Peterson M. Luyckx K. Andrews J. A. (2016). Purpose in life in emerging adulthood: development and validation of a new brief measure. *J. Posit. Psychol.* 11 237–245. 10.1080/17439760.2015.1048817 26958072 PMC4779362

[B35] Hoaglin D. C. Iglewicz B. (1987). Fine-tuning some resistant rules for outlier labeling. *J. Am. Stat. Assoc.* 82 1147–1149. 10.1080/01621459.1987.10478551

[B36] Holsen I. Geldhof J. Larsen T. Aardal E. (2017). The five Cs of positive youth development in Norway: assessment and associations with positive and negative outcomes. *Int. J. Behav. Dev.* 41 559–569. 10.1177/0165025416645668

[B37] Kashdan T. B. McKnight P. E. (2009). Origins of purpose in life: refining our understanding of a life well lived. *Psihologijske Teme* 18 303–313.

[B38] Keyes C. L. (2002). The mental health continuum: from languishing to flourishing in life. *J. Health Soc. Behav.* 43 207–222. 10.2307/3090197 12096700

[B39] Keyes C. L. (2005). Mental illness and/or mental health? Investigating axioms of the complete state model of health. *J. Consult. Clin. Psychol.* 73 539–548. 10.1037/0022-006x.73.3.539 15982151

[B40] Keyes C. L. (2009). *Atlanta: Brief Description of the Mental Health Continuum Short Form (MHC-SF).* 10.1037/t30592-000

[B41] Knofczynski G. T. Mundfrom D. (2008). Sample sizes when using multiple linear regression for prediction. *Educ. Psychol. Meas.* 68 431–442. 10.1177/0013164407310131

[B42] Korkeila J. Lehtinen V. Bijl R. Dalgard O. S. Kovess V. Morgan A. (2003). Establishing a set of mental health indicators for Europe. *Scand. J. Public Health* 31 451–459. 10.1080/14034940210165208 14675937

[B43] Kotera Y. Ting S. H. (2021). Positive psychology of Malaysian university students: impacts of engagement, motivation, self-compassion, and well-being on mental health. *Int. J. Ment. Health Addict.* 19 227–239. 10.1007/s11469-019-00169-z

[B44] Lenz A. S. (2021). Evidence for relationships between hope, resilience, and mental health among youth. *J. Couns. Dev.* 99 96–103. 10.1002/jcad.12357

[B45] Lerner R. M. (2009). “The positive youth development perspective: theoretical and empirical bases of a strengths-based approach to adolescent development,” in *The Oxford Handbook of Positive Psychology*, eds Lopez S. J. Snyder C. R. (Oxford: Oxford University Press), 149–163.

[B46] Lerner R. M. Lerner J. V. Almerigi J. B. Theokas C. Phelps E. Gestsdottir S. (2005). Positive youth development, participation in community youth development programs, and community contributions of fifth-grade adolescents: findings from the first wave of the 4-H study of positive youth development. *J. Early Adolesc.* 25 17–71. 10.1177/0272431604272461

[B47] Li M. Y. Yang Y. L. Liu L. Wang L. (2016). Effects of social support hope and resilience on quality of life among Chinese bladder cancer patients: a cross-sectional study. *Health Qual. Life Outcomes* 14:73. 10.1186/s12955-016-0481-z 27153944 PMC4859956

[B48] Lopez S. J. Snyder C. R. (eds.). (2005). *The Oxford Handbook of Positive Psychology*, 2nd Edn. New York: Oxford University Press.

[B49] Marques S. C. Gallagher M. W. Lopez S. J. (2017). Hope-and academic-related outcomes: a meta-analysis. *School Ment. Health* 9 250–262. 10.1007/s12310-017-9212-9

[B50] Marques S. C. Pais-Ribeiro J. L. Lopez S. J. (2011). The role of positive psychology constructs in predicting mental health and academic achievement in children and adolescents: a two-year longitudinal study. *J. Happiness Stud.* 12 1049–1062. 10.1007/s10902-010-9244-4

[B51] Munoz R. T. Hellman C. M. Brunk K. L. (2017). The relationship between hope and life satisfaction among survivors of intimate partner violence: the enhancing effect of self-efficacy. *Appl. Res. Qual. Life* 12 981–995. 10.1007/s11482-016-9501-8

[B52] Orpana H. Vachon J. Dykxhoorn J. McRae L. Jayaraman G. (2016). Monitoring positive mental health and its determinants in Canada: the development of the positive mental health surveillance indicator framework. *Health Promot. Chronic Dis. Prev. Can.* 36 1–10. 10.24095/hpcdp.36.1.01 26789022 PMC4939463

[B53] Oxford R. L. Burry-Stock J. A. (1995). Assessing the use of language learning strategies worldwide with the ESL/EFL version of the Strategy Inventory for Language Learning (SILL). *System* 23 1–23. 10.1016/0346-251x(94)00047-a

[B54] Park N. (2004). The role of subjective well-being in positive youth development. *Ann. Am. Acad. Polit. Soc. Sci.* 591 25–39.

[B55] Parker P. D. Ciarrochi J. Heaven P. Marshall S. Sahdra B. Kiuru N. (2015). Hope, friends, and subjective well-being: a social network approach to peer group contextual effects. *Child Dev.* 86 642–650. 10.1111/cdev.12308 25327644

[B56] Peterson C. Seligman M. E. (2004). *Character Strengths and Virtues: A Handbook and Classification* (Vol. 1). Oxford University Press.

[B57] Preacher K. J. Hayes A. F. (2008). Asymptotic and resampling strategies for assessing and comparing indirect effects in multiple mediator models. *Behav. Res. Methods* 40 879–891. 10.3758/BRM.40.3.879 18697684

[B58] Rand K. L. Cheavens J. S. (2009). “Hope theory,” in *Oxford Handbook of Positive Psychology*, eds Lopez S. J. Snyder C. R. (Oxford: Oxford University Press), 323–333.

[B59] Ryan R. M. Deci E. L. (2001). On happiness and human potentials: a review of research on hedonic and eudaimonic well-being. *Ann. Rev. Psychol.* 52 141–166. 10.1146/annurev.psych.52.1.141 11148302

[B60] Smith E. P. Osgood D. W. Oh Y. Caldwell L. C. (2018). Promoting afterschool quality and positive youth development: cluster randomized trial of the PAX good behavior game. *Preve. Sci.* 19 159–173. 10.1007/s11121-017-0820-2 28766191 PMC6533071

[B61] Stoyles G. Chadwick A. Caputi P. (2015). Purpose in life and well-being: the relationship between purpose in life, hope, coping, and inward sensitivity among first-year university students. *J. Spiritual. Ment. Health* 17 119–134. 10.1080/19349637.2015.985558

[B62] Tengland P. A. (2001). “Marie Jahoda’s current concepts of positive mental health,” in *Mental Health*, (Dordrecht: Springer), 47–78. 10.1007/978-94-017-2237-7_4

[B63] Tirrell J. M. Geldhof G. J. King P. E. Dowling E. M. Sim A. T. Williams K. (2019). Measuring spirituality, hope, and thriving among salvadoran youth: initial findings from the compassion international study of positive youth development. *Child Youth Care Forum* 48 241–268. 10.1007/s10566-018-9454-1

[B64] Vaillant G. E. (2012). Positive mental health: is there a cross-cultural definition? *World Psychiatry* 11 93–99. 10.1016/j.wpsyc.2012.05.006 22654934 PMC3363378

[B65] Van Dyke C. J. Elias M. J. (2007). How forgiveness, purpose, and religiosity are related to the mental health and well-being of youth: a review of the literature. *Ment. Health Relig. Cult.* 10 395–415. 10.1080/13674670600841793

[B66] World Health Organization [WHO] (2014). *Mental Health: Strengthening Our Response.* Geneva: WHO.

[B67] Yalçın İ Malkoç A. (2015). The relationship between meaning in life and subjective well-being: forgiveness and hope as mediators. *J. Happiness Stud.* 16 915–929. 10.1007/s10902-014-9540-5

[B68] Zhang M. X. Mou N. L. Tong K. K. Wu A. (2018). Investigation of the effects of purpose in life, grit, gratitude, and school belonging on mental distress among Chinese emerging adults. *Int. J. Environ. Res. Public Health* 15 2147–2159. 10.3390/ijerph15102147 30274292 PMC6210347

[B69] Zhang Y. Chen M. (2018). Character strengths, strengths use, future self-continuity and subjective well-being among Chinese university students. *Front. Psychol.* 9:1040. 10.3389/fpsyg.2018.01040 30008686 PMC6034163

[B70] Zhu X. Shek D. T. (2020). Impact of a positive youth development program on junior high school students in mainland China: a pioneer study. *Child. Youth Serv. Rev.* 114:105022. 10.1016/j.childyouth.2020.105022

